# Indigestion of Infants

**Published:** 1891-10

**Authors:** Nathan Cash

**Affiliations:** Uhrichsville, O.; Clinical Bureau, I. H. A.


### INDIGESTION IN INFANTS.

NATHAN CASH, M. D., UHRICHSVILLE, O.

(Clinical Bureau, I. H. A.)

To the officers and members of "The International Hahnemannian Association," greeting.

It becomes my duty to again address you on behalf of suffering humanity and in doing so I wish to call your attention to a subject which, to me, is one of paramount importance to any other in our daily lives—viz., indigestion in infants.

This ailment is much more prevalent in summer than in winter, and the reasons for considering this subject at this time are obvious. You are all probably aware that the same amount of food is not required in warm weather that is demanded in the cold seasons, and for this reason greater care should be taken in the warm seasons to prevent derangement of the digestion of all, but more especially of infants.

We seldom have serious indigestions in infants which are fed at the breast, because the supply is soon regulated to the wants of the child. This is usually the result in mothers of good health and natural ways of living.

The case is quite the reverse in *very* many instances, especially in communities in which fashionable society prevails. The feeding of infants is one of the most important duties of the mother, and demands the attention of the physician equally as much as any other, more especially in the warm months of the year. My experience leads to the conclusion that very few mothers give this subject the attention it deserves, and I fear the same may be said of many physicians. There is no food so suitable for young children as *milk*, nor is there *any* substitute worthy of consideration when milk can be obtained. If milk *can* be obtained nothing else should be given. It matters not whether the milk is obtained from the goat, ass, mare, cow, or woman, so the animal giving it is not sick or diseased. I have no friendship whatever for the various and numerous artificial foods so prominently and persistently offered to the public; and for physicians to allow themselves to be made the means of increasing if not creating a trade for them is reprehensible. Within an hour or two after birth the infant should be put to the breast, and *that* is all it should have, except fresh water, until it is proved that the mother *cannot* give it nourishment. Now is the time to consider the subject of feeding "by hand."

As it has become so fashionable to resort to all kinds of decoctions and compounds and artificial foods for the baby instead of milk, I feel it my imperative duty to enter my emphatic protest. The point we would recommend and insist upon is, when the mother *cannot* or *will not* nurse her babe, to give *good* milk in moderate quantities, and have it as fresh as possible, and if it still retains its animal heat so much the better. I say in moderate quantities, because if more is given than will properly digest, it is the starting point of *indigestion*, the subject of this paper.

There is a great difference in the capacity of digestion and feeding in different individuals, and, as before remarked, the season has great bearing on the feeding capacity.

What would be a moderate meal for one child at one time might endanger the life of another, so that great care should be taken to ascertain the capacity of digestion of the individual under consideration. It should be remembered that the crying of infants is the only natural language, and because a child cries it is no indication that it is because of hunger or pain, and must be fed or drugged; but, on the contrary, a child should not be fed oftener than two to three hours. The simple crying of the infant is not injurious and should not be regarded with such apprehensions of danger or as an indication of extreme pain as to justify the use of any of the numerous preparations of Opium or soothing syrups or cordials; for even very young infants often exhibit strong indications of temper and sometimes a downright fit of anger. Under such circumstances it is quite customary to give something to quiet the child under the supposition that it has the colic; but it is far less injurious to let the child have its cry out than to give any of the opiates. The observant physician will soon distinguish between the cry of pain and that of anger or the want of attention.

We do not advocate neglect of the child by any means when there are indications of pain or other causes of sickness, but we do wish to disabuse the minds of physicians and parents of the injurious effects of crying, to quiet which, some mothers and we fear some physicians would keep a child too drunk to cry, with anything that would produce such a result, no difference how injurious in its after-effects.

We venture the assertion that infant mortality is more than one-half greater because of over-feeding than would be the case if one-half the quantity was given at the proper time and no anodynes given. Or, in other words, more children are fed to death than die of starvation; and, again, more children are drugged to death than die from natural diseases, while those children that survive the pernicious effects of both are little more than wrecks of what they should have been.

It frequently occurs that everything *but* milk is suggested as food for the infant, and if milk is selected it is so doctored as to be impossible of recognition.

We repeat the statement that the proper food for infants is *milk*, MILK! Good, fresh milk, with nothing added nor anything extracted, for Nature's laboratory is superior to the chemist's.

To read the remarks of the manufacturers of the various artificial foods the novice might well be excused for supposing the only thing necessary for the perpetuation of the race would be to haul the materials to the chemist's laboratory; but such is not our experience. These old-fashioned notions and ideas may not please the extra-scientific—those who repudiate the 200th potencies because the microscope reveals nothing above the 12th potency—but they stand the tests at the bedside and sick-room. Even scalded milk or skimmed milk should be excluded, because in either case the butter has disappeared. The milk should be carefully carried from the cow to the child; milk hauled over country roads or city streets is not fit for infant food unless the receptacle is filled as full as it will hold to prevent churning.

One thing more is to be strictly observed, and that is, if anything needs doctoring it is the child and not the milk; although it sometimes becomes necessary to doctor the nurse, the family, or possibly a whole neighborhood before you can have things your own way with the baby. When proper food will not remain on the child's stomach and it is constantly fretting and crying, restless, in most cases diarrhœa, consisting of greenish-yellow stools, foul or sour smelling, vomiting of milk, either curdled or sour, and even water, you may conclude the child has indigestion.

In this condition the question is often asked: "Doctor, with what shall we feed the baby?" Your answer should be, milk. "But, doctor, the milk disagrees with the baby; it won't stay down, or it passes off undigested. We have put lime-water in the milk; we have scalded the milk; we have mixed the milk with water and sugar; we have soaked crackers in it, and we have given Pepsin with the milk; we have given Castoria and Castor oil, and I don't know what we have not done, and still the child don't get any better."

"What else have you fed the baby with all this while?"

"Oh! we have fed it 'Malted Milk,' 'Mellin's Food" 'Imperial Granum,' and a host of other foods, and still it acts as if it was starved," which in reality is the case.

Now is it any wonder the child is sick? It would be a greater wonder if it was not. Still your answer should be, "Milk is the proper food for this baby."

The above picture is not overdrawn, for just such cases are found all through the warm seasons in and out of the cities. The duty of the physician in such cases is to first point out the errors, and next instruct the mother how to manage the feeding as well as to apply the remedies to correct these conditions.

The errors she has committed are: First, she has fed the babe too much; second, she did not let the child go long enough to allow the stomach to rest and recover, but kept on feeding, thereby adding fuel to fire; next you went to doctoring the milk, by putting lime-water into it and adding sugar and water, teas and toddy, and Pepsin. Then you resorted to the anodynes to quiet its cries, which was doctoring the child in the wrong direction. The child, it is true, needed the greatest attention, but to stupefy a child with any form of Opium or narcotic is not the proper way to make a healthy baby, because it is only a palliative and is bound to be disastrous. Rest is what this child needs. Rest from feeding, except fresh water, for eight to ten hours at the first start. Rest from everything whatever, but very small quantities of fresh, warm milk and fresh water later on for several days. Rest from " Castoria," Castor oil, " Godfrey's Cordial," " Winslow's Soothing Syrup," etc., forever. Your lime-water added constipation, and on that account should be excluded also. These are heroic measures, but they must be adopted if you expect to make a success in treating infants with this trouble and rescue them from an early grave; for the majority of such cases are called cholera infantum, while in reality they are nothing more than indigestion. Good fresh water plays an important part in the treatment of this affection; water fresh from the spring, well, or hydrant. Ice-water should be strictly forbidden. Give the child all the water you can induce it to swallow for two or three days. Much firmness will be necessary, but a community will soon be convinced, by your success, that you have good grounds for the faith and courage you manifest, and your labor will become easier year by year.

I will now speak of the medicinal means to be used for these cases. The list need not be long for the purpose of this paper, still they are of the greatest importance. I will mention but eight remedies, viz., Æthusa-cyn., Arsen., Bry., Calc-c., Cham., Nux-vom., Podo., and Puls., but, above all, I would call your attention to Æthusa-c.

While you have the whole materia medica to choose from for particular cases, we would not have you think I have undue partiality for any one remedy or am guilty of routine practice. I will therefore give the leading pathogenetic symptoms of Æthusa-c. applicable to those stomach and bowel troubles.

See Hering's *Guiding Symptoms.* Spasmodic hiccough, empty eructations, violent sudden vomiting, vomiting of milk-white substance, vomiting of yellow fluid followed by curdled milk and cheesy matter, vomiting of greenish phlegm similar to the stools.

The milk is forcibly ejected soon after taking. Profuse vomiting of water, copious greenish vomiting, pains in the stomach accompanied by fearful vomiting, cramps in the stomach, excessive griping pains in the belly.

Colic with diarrhœa, excessive griping pains in the abdomen, stools of partly digested food, diarrhœa; discharges green, thin, bilious, with violent tenesmus. Bright yellow or greenish, watery, slimy stools, with crying and drawing up of the feet. Evacuations of thin, bright yellow or greenish fluid mixed with much bile, with severe tenesmus.

Most obstinate constipation with feeling as if all action of the bowels had been lost. Thirst, with total loss of appetite for every kind of aliment. Burning thirst, intolerance of milk. Aphthæ in the mouth and throat.

A drawn condition (of the muscles of the mouth), beginning at the alæ nasi, and extending to the angles of the mouth, gave the face an expression of great anxiety and pain. The features have an expression of great anguish and pain. Great agitation, anxiety, and restlessness, bad humor, irritability, morose and cross. Great nervousness, constant anxiety and weak feeling, lies unconscious, dilated pupils, staring eyes.

As to the manner of using the remedy, I generally give it in water, one teaspoonful of the solution every hour or two until I get the vomiting arrested, then every two or three hours for twelve or fourteen hours, then give nothing but Sac-lac., except milk or fresh water for a day or two, thus giving me a chance to see what it needed further.

As to potency I use the 30th, 2C, 5C, 1M, 50M, and CM. Wine aggravates nearly all symptoms of Æthusa-c. I have made little effort at arrangement, but simply put my thoughts on paper as they came. Each one can arrange the material to suit himself. The effort has been to impress the main points on your minds, and if this paper should help others to manage successfully those troublesome cases the writer will be amply compensated.

